# Supplementary material for: Causes and consequences of sex-chromosome turnovers in Diptera
Source: Evol Lett. 2026 Mar 12;10(3):253–68. doi: 10.1093/evlett/qrag003 (PMC13231274; doi:10.1093/evlett/qrag003)
Supplement: qrag003_Supplemental_Files [file qrag003_supplemental_files.zip › Supplementary_Datasets.zip!/Supplementary_Datasets/Dataset_2_Graphical_representation_outgroups/Syrphidae_Epistrophella_euchroma_chromosomes.pdf]

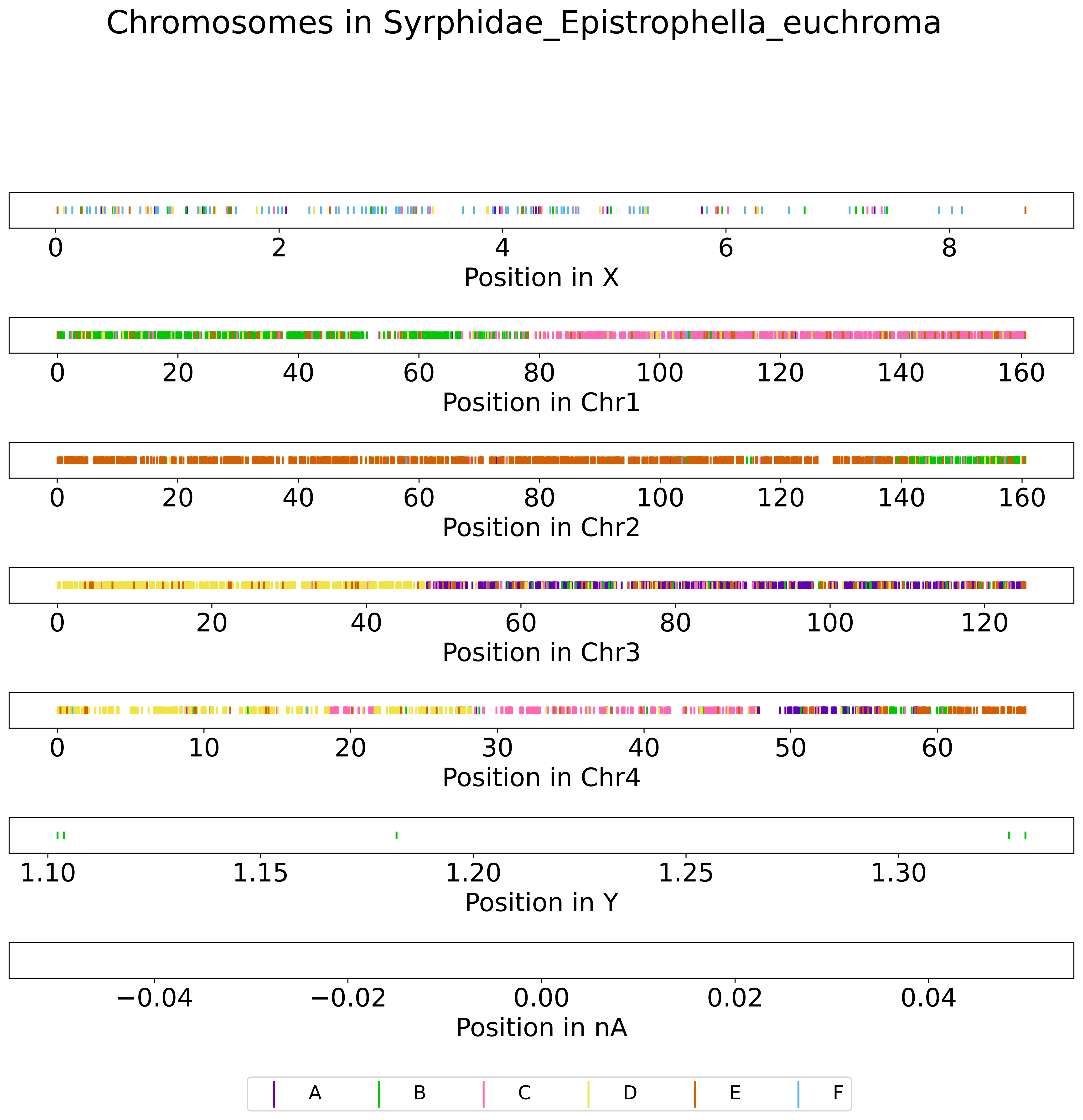

Chromosomes in Syrphidae_Epistrophella_euchroma
0
2
4
6
8
Position in X
0
20
40
60
80
100
120
140
160
Position in Chr1
0
20
40
60
80
100
120
140
160
Position in Chr2
0
20
40
60
80
100
120
Position in Chr3
0
10
20
30
40
50
60
Position in Chr4
1.10
1.15
1.20
1.25
1.30
Position in Y
−0.04
−0.02
0.00
0.02
0.04
Position in nA
A
B
C
D
E
F
